# Uterine lesions with sex cord-like architectures: a systematic review

**DOI:** 10.1186/s13000-019-0909-y

**Published:** 2019-11-18

**Authors:** Meng Jia, Ping-Li Sun, Hongwen Gao

**Affiliations:** Department of pathology, The Second Hospital of Jilin University, Changchun, Jilin, 130041 China

**Keywords:** UTROSCT, Endometrial stromal tumor, Sex cord, Uterine, Immunohistochemistry

## Abstract

**Background:**

Sex cord-like elements are rarely observed in uterine lesions, but these morphological patterns could appear in a variety of uterine tumors and non-tumorous lesions. In this review, we collected the literatures regarding the uterine tumorous and non-tumorous lesions containing sex cord-like elements and summarized these lesions in terms of clinicopathological, immunohistochemical, and molecular features in order to further understand these lesions and provide some new ideas for differential diagnosis.

**Main body:**

This section provides a comprehensive overview of the clinicopathological, immunohistochemical, and molecular features of uterine lesions with sex cord-like architectures including uterine tumors resembling ovarian sex cord tumors, endometrial stromal tumors, adenomyosis, endometrial polyps, leiomyoma, epithelioid leiomyosarcoma, adenosarcoma, sertoliform endometrioid carcinoma, corded and hyalinized endometrioid carcinoma, mesonephric adenocarcinoma, and mesonephric-like adenocarcinoma. The differential diagnosis based on morphology, immunohistochemistry, and molecular alterations has also been discussed.

**Conclusion:**

The sex cord-like areas in these lesions show heterogeneous but similar morphological features. Additionally, immunohistochemical staining plays a limited role in differential diagnosis. Furthermore, it is of significance for pathologists to better understand these lesions in order to avoid confusion and mistakes during pathological diagnosis, especially in a biopsy/curettage specimen.

## Background

Sex cord-like elements are rarely observed in uterine lesions, but these morphological patterns could indeed appear in a variety of uterine tumors and non-tumorous lesions. In 1976, Clement and Scully [1] described two uterine tumor groups with histological resemblances to ovarian sex-cord tumors: Group I consists of typical endometrial stromal tumors (EST) with a sex cord-like contribution as a minor component (10 to 40%) and is mainly composed of endometrial stromal nodules (ESN) and low grade endometrial stromal sarcomas (LGESS) [2], also known as endometrial stromal tumors with sex cord-like elements (ESTSCLE); Group II consists of tumors formed predominantly or exclusively by a sex cord-like component, known as uterine tumor resembling ovarian sex cord tumors (UTROSCT). Except UTROSCT and ESTSCLE, other uterine lesions with morphological features resembling sex cord-like architectures include adenomyosis [3], endometrial polyp [4], leiomyoma [5], epithelioid leiomyosarcoma [6], adenosarcoma (AS) [7–12], sertoliform endometrioid carcinoma [13–16], corded and hyalinized endometrioid carcinoma (CHEC) [17], mesonephric and mesonephric-like adenocarcinoma [18–21], and so on. The sex cord-like elements in these lesions may cause confusion and mistakes during pathological diagnosis, especially in a biopsy/curettage specimen.

To the best of our knowledge, few articles have summarized the clinicopathological features and prognosis of uterine lesions with sex cord-like architectures. Meanwhile, although UTROSCT has been reported in numerous articles, the molecular profile of this tumor has never been summarized comprehensively, and the differences between this tumor and the sex cord-like elements in other uterine lesions have been poorly described, both morphologically and immunohistochemically. Therefore, in this review, we collected the literatures regarding the uterine tumorous and non-tumorous lesions containing sex cord-like elements, and summarized these lesions in terms of clinicopathological, immunohistochemical, and molecular features in order to further understand these lesions and provide some new ideas for differential diagnosis.

### Main text

This section provides an overview of the clinicopathological, immunohistochemical, and molecular features of 10 categories of uterine lesions with sex cord-like architectures. Among these lesions, UTROSCT and EST have been studied systematically in a few articles, whereas the other lesions are described dispersedly. The clinicopathological features and immunohistochemical profile of the cases with sex cord-like elements, except UTROSCT and EST, are summarized in Table 1 and Table 2.

**Table 1:** Clinicopathological features of uterine tumors with sex cord-like architectures in literatures

Reference	Category	Number of cases	Age	Size (cm)	Location	Tamoxifen usage	Sex cord-like propotion	Histological features	FIGO Stage	Accompanied diseases	Follow-up
Fukunaga M [3]	Adenomyosis	1	43	0.3	NA	NA	NA	NA	NA	Leiomyomas, an adenomatoid tumor and ovarian endometriotic cysts	36 months ANED
Stewart CJ [22]		1	52	1.2	NA	NA	40%	NA	NA	NA	NA
De Quintal MsM [4]	Endometrial polyp	1	63	3	Uterine fundus	Present	NA	NA	NA	Adenomyosis	NA
Pusiol T [5]	Leiomyoma	2	55, 64	3.5, 1.9	Submucosal, left wall of the uterus	NA	Diffuse	NA	NA	NA	NA
Lee FY [6]	Epithelioid leiomyosarcoma	1	31	NA	NA	NA	NA	The tumor cells showed prominent nucleoli and high Ki-67.	NA	NA	NA
Murray SK [17]	CHEC	31	25–83 (mean 52)	NA	NA	NA	10–90%	Grade 1(35.5%) Grade 2(64.5%) Squamous differentiation were present in 70% of the cases.	IA(63.0%) IB(11.1%) II(18.5%) IIIC(3.7%) IV(3.7%)	NA	2–115 months(mean 34.4 months) 83.3%ANED 5.6%DOD 5.6%DOC 5.6%AWD
Wani Y [23]		6	38–57 (mean 46)	NA	NA	NA	Less than 5–40%	Squamous differentiation were present in 83.3% of the cases.	NA	NA	NA
Fox H [13]	Sertoliform endometrioid adenocarcinoma	1	41	2	Upper left side of the uterine cavity	NA	NA	NA	IB	NA	NA
Usadi RS [14]		1	62	7	Anterior endometrium	NA	NA	No squamous differentiation was seen.	II	NA	NA
Eichhorn JH [15]		4	44–83 (mean 64.5)	1.7–5(mean 3.9)	Two cases located in the uterine fundus.	NA	10%-more than 85%	Squamous differentiation were present in 3 cases.	IA(25.0%) IB(75.0%)	Leiomyomas, adenomyosis and endometrial hyperplasia	NA
Liang SX [16]		1	71	4.5	Endometrial cavity	NA	More than 80%	Grade 3 No squamous differentiation was seen.	II	Atypical complex endometrial hyperplasia	6 months ANED
Clement PB [7]	Adenosarcoma	8	22–85 (mean 41)	1.5–6	NA	NA	5–50%	Polygonal cells were present.	IB(50.0%) Others were not mentioned	Leiomyomas in 2 cases	3–11 years 85.7% ANED 14.3% DOC
Stolnicu S [9]		2	64, 71	2.5, 8	NA	Present in one case.	More than 75%	Squamous metaplasia was seen in the gland area. Polygonal cells were present.	NA	NA	3 and 5 years ANED
Ulker V [11]		1	47	4	Uterine fundus and cervix	NA	20%	Polygonal cells were present.	IB	NA	2 years ANED
Wu RI [12]		1	28	Multifocal	Uterine and ileum	NA	NA	The stroma showed edematous and hypocellular.	NA	NA	17 months ANED
Mohammadizadeh F [8]		1	31	Multifocal	NA	NA	85%	Polygonal cells were present.	NA	Leiomyomas	NA
Stolnicu S [10]		6	39–71	2.5–19	Four intracavitary, 1 isthmic and 1 ovarian	Present in 2 cases.	60–90%	Squamous metaplasia was seen in the gland area. Polygonal cells were present.	I	NA	26–102 months ANED
Stewart CJ [22]		3	35–70	3–8	NA	NA	10–20%	NA	NA	NA	NA
Yamamoto Y [20]	Mesonephric adenocarcinoma	1	58	14	Left lateral wall of the uterine body	NA	NA	The tumor contained sarcomatous components. Stromal hyalinization was present.	IB	NA	8 months DOD
Wu H [19]		2	55, 62	3.5, 8	Lower 1/3 portion lateral wall of the uterus, higher 2/3 portion of the uterus	NA	NA	The tumor contained sarcomatous components.	IB	NA	Less than 1 months and 7 months ANED
Na K [18]		4	53–70	2.2–4.3	NA	NA	NA	Two cases contained sarcomatous components.	IA(25.0%) IB(50.0%) IIIB(25.0%)	NA	7–20 months 75.0%ANED 25.0%AWD
Patel V [21]	Mesonephric-like adenocarcinoma	1	71	3	Endometrium	NA	30%	No squamous or mucinous differentiation or associated mesonephric remnants was seen.	NA	NA	NA

Abbreviations: *ANED* alive with no evidence of disease; *AWD* alive with disease; *CHEC* corded and hyalinized endometrioid carcinoma; *DOC* died of other causes; *DOD* dead of disease; *NA* not available

**Table 2:** Immunohistochemical profile of uterine tumors with sex cord-like architectures (sex cord-like area) in literatures

Reference	Category	cytokeratin	EMA	vimentin	ER	PR	CD10	WT1	Desmin	SMA	calretinin	inhibin	CD56	melan A	CD99	SF-1	FOLX2	Ki67
Fukunaga M [3]	Adenomyosis	–	NA	+	+	+	NA	NA	+(dot-like)	+	NA	–	NA	NA	NA	NA	NA	NA
Stewart CJ [22]		–	NA	NA	+	+	–	NA	+	+	–	–	+	NA	–	–	–	NA
De Quintal MsM [4]	Endometrial polyp	+	–	+	NA	NA	NA	NA	+(focally)	NA	NA	–	NA	NA	+	NA	NA	NA
Pusiol T [5]	Leiomyoma	+(weakly)	NA	NA	+	+	–	NA	NA	NA	–	–	NA	–	–	NA	NA	NA
Lee FY [6]	Epithelioid leiomyosarcoma	NA	NA	+	NA	NA	–	+	+	+	–	–	NA	NA	+	NA	NA	High
Murray SK [17]	CHEC	+(13/16)	NA	+(14/16)	+(5/10)	NA	–	NA	–	–	NA	–	NA	NA	NA	NA	NA	NA
Usadi RS [14]	Sertoliform endometrioid adenocarcinoma	+	+	NA	NA	NA	NA	NA	NA	NA	NA	NA	NA	NA	NA	NA	NA	NA
Eichhorn JH [15]		+	+	+	NA	NA	NA	NA	–	–	NA	NA	NA	NA	NA	NA	NA	NA
Liang SX [16]		–	+	+(focally)	+	+	–	+(focally)	–	–	+(focally)	+	NA	+(focally)	+	NA	NA	NA
Stolnicu S [9]	Adenosarcoma	+	NA	NA	NA	+	–	NA	–	NA	+	+	+	NA	NA	NA	NA	NA
Ulker V [11]		NA	NA	NA	NA	NA	+	NA	NA	NA	+	+	NA	NA	NA	NA	NA	NA
Wu RI [12]		–	–	NA	+	+	+(patchy)	NA	+	NA	+	+	NA	–	NA	NA	NA	< 5%
Mohammadizadeh F [8]		+(focally)	NA	+	+	+	–	–	NA	–	+	NA	–	–	+	NA	NA	2%
Stolnicu S [10]		+	NA	+	NA	+(variably)	–	+	+(variably)	NA	+	+	+	NA	–	NA	NA	NA
Stewart CJ [22]		+	NA	NA	+	+(2/3)	+(2/3)	NA	+(2/3)	+(1/3)	+(1/3)	–	+(1/3)	NA	+(1/3)	–	+(2/3)	NA
Patel V [21]	Mesonephric-like adenocarcinoma	NA	NA	NA	–	–	NA	NA	NA	NA	NA	NA	NA	NA	NA	NA	NA	NA

Abbreviations: *CHEC* corded and hyalinized endometrioid carcinoma; *NA* not available

### UTROSCT

**Fig. 1:**
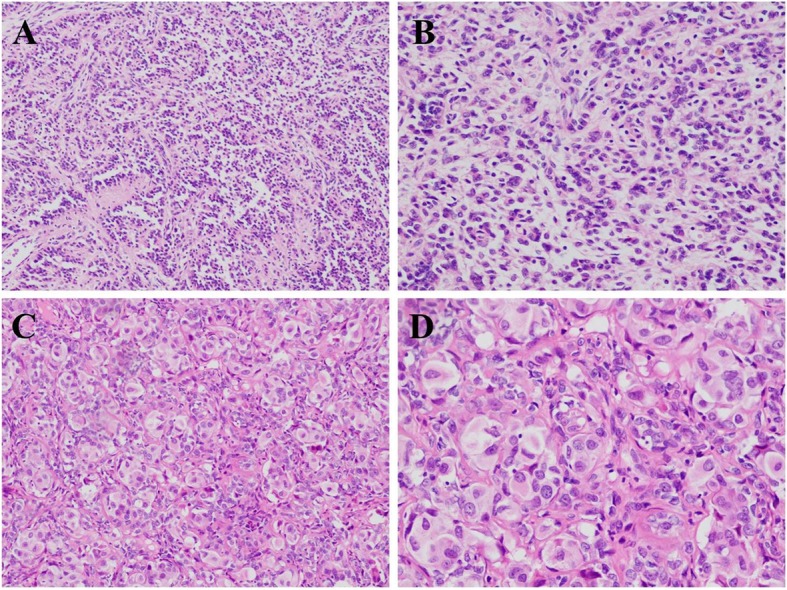
The representative pictures of UTROSCT. (**a**) intermediate magnification; (**b**) high magnification; (**c**, **d**) UTROSCT with polygonal cells (Hematoxylin-eosin staining, **a**, **c**: × 100, **b**, **d**: × 200)

UTROSCT is a rare tumor with predominantly morphological features of sex cord-like elements wherein tumor cells arrange in cords, trabeculaes, tubules, clusters, sheets, and retiform appearance. Tumor cells in UTROSCT show two features. In most cases, the tumor cells are of small to medium size and oval to spindle in shape, with mild to moderate cell atypia, scanty cytoplasm, and an unobvious nucleolus, similar to the cells of the adult granulosa cell tumors (Fig. 1a-b). In other cases, the tumor cells are larger in size with a prominent foamy or eosinophilic cytoplasm and an irregular nucleus, resembling foam cells or macrophages (intranuclear vacuoles and nucleolus can also be observed) (Fig. 1c-d). This cellular type is the so-called “polygonal cells with eosinophilic or foamy cytoplasm,” resembling Sertoli cells [24]. Immunohistochemically, UTROSCT characteristically exhibits a polyphenotypic immunophenotype with co-expression of hormone receptors, cytokeratin, smooth muscle markers, and markers that are commonly positive in ovarian sex cord–stromal tumors including, inhibin, calretinin, CD56, CD99, Melan-A, steroidogenic factor-1 (SF-1), and FOXL2 [22, 25].

The diagnosis of UTROSCT has been discussed based on immunohistochemical expression. In some articles, positive staining of calretinin is regarded as necessary, which is thought to be the most specific marker of this tumor [26–28], however, calretinin-negative UTROSCT has also been reported [25, 29]. In contrast, Stewart et al. [22] regarded the positive staining of SF-1 as a useful indication for the differential diagnosis of UTROSCT and other uterine lesions with sex cord-like architectures; according to their study, SF-1 showed a specificity of 100.0%. In UTROSCT, the expression of SF-1 was studied in two articles containing 19 and 6 cases respectively, with positive rates of 57.9 and 50.0% [22, 30], respectively. The specific expression of SF-1 in UTROSCT could be a useful method for differential diagnosis; however, the low expression rate of this marker in UTROSCT might be a limitation.

Molecular changes in UTROSCT have also been reported in a few articles. Although UTROSCT demonstrates FOXL2 protein positivity, FOXL2 and DICER1 mutations are not identified in this tumor [30, 31]. It lacks the IgH gene translocation as well as IgH-Bcl-2, IgH-MALT1 and API2-MALT1 translocations [32]. Meanwhile, UTROSCT is found to contain the t(X;6)(p22.3;q23.1) and t(4;18)(q21.1;q21.3) translocations [33], as well as ESR1-NCOA2/3, GREB1-NCOA1/2 and GREB1-CTNNB1 fusions [34–37]. Among these molecular alterations, ESR1-NCOA3 fusion is predominantly observed. The characteristic ESR1 or GREB1 rearrangement in UTROSCT might be more useful for pathological diagnosis. Furthermore, unlike EST, UTROSCT lacks JAZF1-JJAZ1 translocation [2] and JAZF1 breakapart [38], and this observation supports the hypothesis that UTROSCT is a distinct entity compared with EST.

Recently, Lee et al. described four uterine sarcomas containing the GREB1 fusion genes [36]. Morphologically, these tumors showed, at most, limited sex cord-like features along with fascicular spindle cellular areas resembling monophasic synovial sarcomas. Sex cord markers (calretinin, α-inhibin and Melan-A) were expressed in only one case and the definite pathological classification of these tumors was uncertain. Based on the present results and previous cases, Lee et al. [37] suspected that GREB1-rearranged tumors might include a distinct variant of UTROSCT with a tendency toward the poorly differentiated end of the spectrum, compared with the classic ESR1-rearranged UTROSCT. These findings provide new ideas regarding the molecular classification of UTROSCT. However, as the number of cases involved in this research is limited, further investigation is required.

### EST

**Fig. 2:**
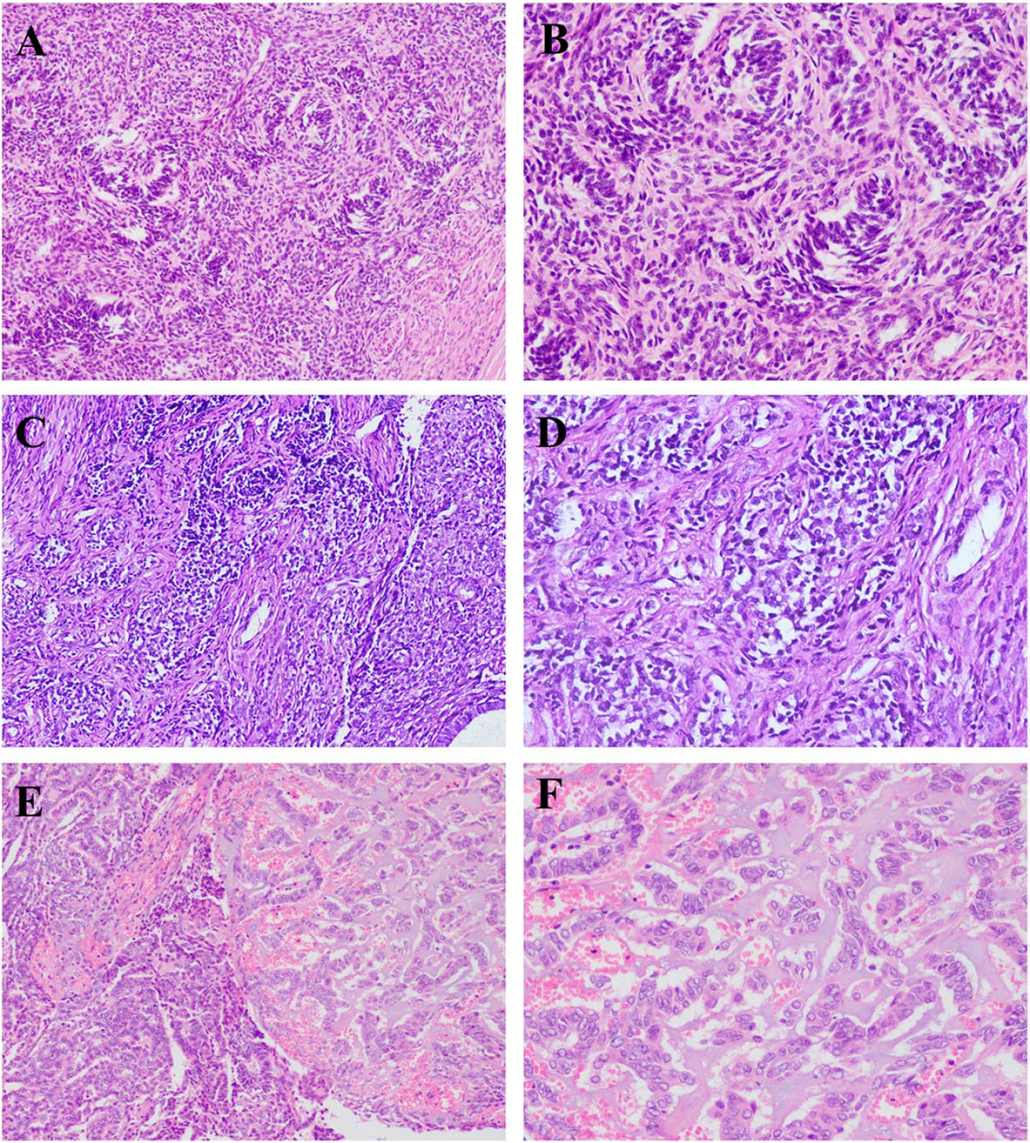
Hematoxylin-eosin staining pictures of LGESS, AS and CHEC. (**a**-**b**) sex cord-like elements in LGESS; (**c**-**d**) sex cord-like elements in AS; (**e**-**f**) sex cord-like elements in CHEC (Hematoxylin-eosin staining, **a**, **c**, **e**: × 100, **b**, **d**, **f**: × 200)

ESN and LGESS could also show sex cord-like features [38–40]. According to Clement and Scully, this variant usually comprises sex cord-like elements below 50% [1], however, LGESS with a sex cord-like contribution more than 50% has also been reported [41, 42]. The tumor cells in the sex cord-like elements are small and uniform and have round or oval nuclei with inconspicuous nucleoli and scanty cytoplasm (Fig. 2a-b). Additionally, nuclear atypia and mitotic activity are not prominent [41, 43]. Moreover, large foam-like or Sertoli-like cells with abundant cytoplasm have also been reported [1, 42, 43]. Immunohistochemically, the sex cord elements show positive staining of epithelial, endometrial stromal, smooth muscle markers and hormone receptors, while epithelial membrane antigen (EMA) is always negative. Sex cord markers including, inhibin (7/30, 23.3%), CD99 (9/19, 47.4%), calretinin (7/20, 35.0%) and CD56 (4/4, 100.0%) could also be positive, while Melan-A (0/3), SF-1 (0/3) and FOXL2 (0/3) are negative [22, 26, 41–47]. Molecularly, ESN with sex cord features could contain JAZF1 breakapart, and LGESS with sex cord features could contain JAZF1, PHF1, EPC1, JAZF1-PHF1, JAZF1-JJAZ1 or EPC1-PHF1 rearrangement [38, 40, 48]. Among these genes, PHF1 rearrangement has been found to be predominant in the sex cord variant of LGESS [40].

### Adenomyosis

Rarely, the stromal element of adenomyosis could show sex cord-like appearance. Based on a case reported by Fukunaga [3], in one focus of adenomyosis, the cells of the sex cord-like area encircled a dilated endometrial gland and were arranged in cords and trabeculaes without prominent cell atypia. Immunohistochemical staining showed that these cells were positive for smooth muscle actin (SMA), desmin, estrogen receptor (ER), progesterone receptor (PR), vimentin and CD56, but negative for CK (AE1/AE3), CAM5.2, CD10, calretinin, inhibin, SF-1, FOXL2, and CD99 [3, 22].

### Endometrial polyp and adenomyoma

The stromal element of endometrial polyps and adenomyoma could show sex cord-like architectures [4, 49]. In these cases, the cells in the sex cord-like area arranged in cords, trabeculaes and sertoliform tubules, and could also be surrounded by hyalinized stroma. These cells showed no mitoses or cellular atypia, and had scanty cytoplasm and bland nuclei. Furthermore, the cells of the sex cord-like area in the endometrial polyp case were reported to be positive for CK (AE1/AE3), CD99 and vimentin, focally positive for desmin, and negative for EMA and inhibin.

### Leiomyoma and leiomyosarcoma

Two leiomyomas with sex cord-like features have been reported in one research article [5] and both cases were well-circumscribed. Tumor cells were arranged in cords and tubules and formed gland-like structures, which were plump but with indistinct cytoplasm and nuclear pleomorphism. Fascicles of smooth muscle cells were also observed. Immunohistochemically, smooth muscle elements and tubular structures were weakly positive for CK (AE1/AE3) and CAM5.2, and the latter component was positive for ER and PR but negative for calretinin, inhibin, CD99, CD10, and Melan-A. The authors of this article named this rare variant “leiomyoma with tubules.” This tumor and another variant of leiomyoma, vascular plexiform leiomyoma [50], showed similar morphological features to UTROSCT. Immunohistochemistry should therefore be used for differential diagnosis.

Epithelioid leiomyosarcoma was also reported to contain sex cord-like elements [6]. In a case reported by Lee et al. [6], besides typical epithelioid leiomyosarcoma area, tumor cells formed tubule-like and cord-like structures and infiltrated the hyalinized and sclerotic uterine stroma. The cells in this area exhibited enlarged oval nuclei, coarse chromatin, and some prominent nucleoli. Immunohistochemical staining was strongly positive for desmin, SMA, CD99, vimentin, WT-1 and a high Ki-67 index, and negative for α-inhibin, calretinin, CD10, and HMB45.

### CHEC

Endometrioid carcinoma with sex cord-like formations and hyalinization is an extremely rare histological subtype of endometrial endometrioid carcinomas, which was first mentioned in a review written by Clement and Young [51]. In 2005, Murray et al. named this subtype “corded and hyalinized endometrioid carcinoma (CHEC)” [17]. With regards to CHEC, only two articles (written in English) were retrieved from PubMed, including 31 cases reported by Murray et al. [17] and 6 cases reported by Wani et al. [23]. Remarkably, CHEC tended to arise in younger patients compared with conventional tumors. Histologically, the features of this variant were characterized by an appearance of 2 components, the conventional endometrioid carcinoma component and sex cord-like component with hyalinization (Fig. 2e-f). The sex cord-like elements in this unique form characterized by bland, epithelioid, fusiform, or spindled cells arranged in cords within a hyalinized stroma. Increased squamous differentiation or morular metaplasia was observed. As for immunohistochemical staining, tumor cells in the areas resembling sex cord-like formations with hyalinization showed a different expression pattern from conventional adenocarcinoma. CK (AE1/AE3) and vimentin were positive in most cases, and although vimentin usually showed diffuse positivity, CK (AE1/AE3) was focally expressed. ER was focally positive in about half of the cases and p53 overexpression was rarely observed. Nuclear expression of β-catenin was noted in spindle and corded cells of sex cord-like areas, while desmin, SMA, inhibin, CD10 and membranous E-cadherin were negative. Molecularly, sequence analysis showed mutations in the exon 3 of β-catenin gene in the areas of sex cord-like formations [23]. Follow-up research revealed that the prognosis of this variant was similar to the conventional endometrioid carcinoma.

### Sertoliform endometrioid carcinoma

Sertoliform endometrioid carcinoma of the endometrium is a rare tumor which contains conventional endometrioid adenocarcinoma elements and areas resembling Sertoli and Sertoli-Leydig cell tumors, and rarely granulosa cell tumors [13–16]. In the latter pattern, tumor cells were arranged as small hollow tubules, cords and trabeculaes, and tightly packed nests. As for cellular features, these cells were columnar with pale oval vesicular nuclei and prominent nucleoli, and apical eosinophilic or clear cytoplasm. Unlike CHEC, this pattern was present between benign or carcinomatous glands without formation of multiple nodular structures or predominant hyalinization. Immunohistochemically, the sertoliform elements were always positive for EMA and negative for smooth muscle markers, which indicated the epithelial nature of these cells. Interestingly, α-inhibin was reported to be positive in one case [16]; however, due to the rarity of this tumor, further study is needed to explore the significance of this expression.

### AS

The stromal elements of Müllerian AS in the uterine corpus, cervix, and ovary could be present with sex cord-like formations or overgrowth, which has been reported in several articles [7–12, 22, 52]. Tumor cells in the sex cord-like areas arranged in cords and tubules could show bilateral cellular morphological features. In some cases, the cells were oval to slightly spindle, with oval nuclei and small nucleoli (Fig. 2c-d); while, large polygonal cells with abundant clear or foamy to eosinophilic cytoplasm could also be observed. The sex cord-like areas could be extensive overgrowth in some cases [8–10]; based on the present data, the massive sex cord-like component was not considered as sarcomatous overgrowth and the patients with this feature did not show worse prognosis. Immunohistochemical staining showed that the sex-cord like elements were α-inhibin and calretinin positive in most cases. Other markers including CK(AE1/AE3), CAM 5.2, vimentin, ER, PR, desmin, SMA, CD10, WT-1, CD56, CD99, and FOXL2 have been observed or reported positive in this area, and the Ki-67 index was varied. Molecularly, ESR1-NCOA2 rearrangement was detected in one case [53].

### Mesonephric adenocarcinoma

Mesonephric adenocarcinoma of the uterine corpus is a rare entity, which originates from mesonephric remnants. This tumor could contain epithelial component only or both epithelial and sarcomatous components. The epithelial component of mesonephric adenocarcinoma could show a variety of architectural patterns, including tubular, glandular, papillary, retiform, glomeruloid, comedonecrosis-like, and sex cord-like patterns [18]. To the best of our knowledge, the sex cord-like pattern of the epithelial component has been reported in seven cases within three articles [18–20]. The sex cord-like pattern consisted of branching cords and trabeculae of tumor cells, which could be separated by acellular, myxoid, hyalinized, or edematous stroma containing small, arborizing blood vessels. Immunohistochemically, researchers reported that tumor cells generally demonstrated positive GATA3, CD10 and PAX2 staining, and negative ER and PR expression; calretinin could be deceptively positive. Molecularly, KRAS, ARID1A, AKT1, CSF1R, GNAQ, NOTCH1, PTCH2, PTEN, ABL1, EPHB4, ATM, RET, CDH1, NF1, MET and ATRX mutations have been found in mesonephric adenocarcinomas. KRAS, ARID1A, ABL1, ATM, RET, and CDH1 mutations have been detected in the mesonephric adenocarcinomas containing sex cord-like pattern; among these genes, KRAS mutation was the most frequent.

### Mesonephric-like adenocarcinoma

Mesonephric-like adenocarcinoma is a newly described entity that is suspected to be a neoplasm exhibiting dual mesonephric and endometrioid differentiation, or alternatively derived from the Müllerian epithelia, but demonstrating predominantly mesonephric differentiation [54, 55]. This tumor predominantly involves the endometrium and shows absence of normal or hyperplastic mesonephric remnants compared with mesonephric adenocarcinoma. The morphological and immunohistochemical features of this tumor are similar to mesonephric adenocarcinoma. Genetically, KRAS and PIK3CA mutations have been detected in uterine mesonephric-like adenocarcinomas, while PTEN, TP53, ARID1A, ARID1B, or SMARCA4 alterations were not detected. Recently, a case of mesonephric-like adenocarcinoma resembling CHEC was reported [21]. This tumor contained areas of stromal hyalinization embedded by tumor cells arranged in cords and trabeculae, which showed low grade cellular atypia. In this area, thyroid transcription factor-1, ER, PR, PAX8, and β-catenin were negative and GATA3 was the only positive marker. KRAS mutation was also detected in this case.

### Differential diagnosis of uterine lesions with sex cord-like architectures

In this brief review, we reviewed the morphological, immunohistochemical, and molecular features of uterine lesions with sex cord-like architectures. The histological characteristics in the sex cord-like areas among these lesions are quite similar and heterogeneous. Although the Sertoli-like polygonal cells are exclusively present in UTROSCT, EST, and AS, this observation has little significance with regard to differential diagnosis. In fact, if the sex cord-like element is observed in a tumor that is totally resected in a tumorectomy or hysterectomy specimen, the diagnosis will not be difficult due to the existence of the conventional parts. However, if the sex cord-like element is the only observation in a biopsy/curettage specimen, it might be impossible to make an exact diagnosis based on morphology.

As for immunohistochemical expression, according to the present results, immunohistochemical staining shows a few differences in the sex cord-like cells among different tumors. However, accurate differential diagnosis by immunohistochemistry is not reliable either. As mentioned above, SF-1 might play an effective role in distinguishing UTROSCT from other lesions, but the low expression rate of this marker in UTROSCT might limit this usage. Calretinin and inhibin might be helpful in distinguishing a small part of these tumors such as UTROSCT, EST, AS and sertoliform endometrioid adenocarcinoma, however, as the positive rate of these markers varies, a negative immunohistochemical result cannot rule out the diagnosis. Especially in EST, the positive rate of calretinin or inhibin is generally low. The immunohistochemical features of uterine lesions with sex cord-like architectures has not been well illuminated because of the rarity of these lesions, and considering the present data, immunohistochemistry might be helpful, but not decisive.

Compared with immunohistochemistry, molecular detection seems more promising regarding differential diagnosis of certain tumors. As mentioned above, the related translocation genes in UTROSCT and EST are different, and the other tumors such as CHEC and mesonephric-related adenocarcinoma, contain characteristic genetic alterations. Interestingly, one AS has been found to contain the same ESR1-NCOA2 rearrangement as reported in UTROSCT. As the number of cases is quite low, however, the significance of this observation needs further study. Molecular features of uterine lesions with sex cord-like architectures remain largely unknown; considering molecular tests are expensive and time consuming and the sex cord-like lesions are rarely observed, it may take time to acquire a full view of their molecular profiles.

## Conclusion

Overall, we reviewed the literatures about the uterine tumorous and non-tumorous lesions containing sex cord-like elements and summarized these lesions in terms of clinicopathological, immunohistochemical and molecular features. Sex cord-like elements are rarely observed in uterine lesions, but these morphological patterns could indeed appear in a variety of these lesions. Additionally, according to our review, immunohistochemical staining plays a limited role in differential diagnosis. Above all, it is of significance for pathologists to acquire a better understanding of these lesions in order to avoid confusion and mistakes during pathological diagnosis, especially in biopsy/curettage specimens.

## Data Availability

The datasets used and/or analyzed during the current study are available from the corresponding author on reasonable request.

## Abbreviations

AS: Adenosarcoma
CHEC: Corded and hyalinized endometrioid carcinoma
ER: Estrogen receptor
ESN: Endometrial stromal nodule
EST: Endometrial stromal tumor
ESTSCLE: Endometrial stromal tumors with sex cord-like elements
LGESS: Low grade endometrial stromal sarcoma
PR: Progesterone receptor
SF-1: Steroidogenic factor-1
SMA: Smooth muscle actin
UTROSCT: Uterine tumor resembling ovarian sex cord tumor
